# Quantitative assessment of emphysema and bronchial wall thickness in patients with stable chronic obstructive pulmonary disease: comparison between the eosinophilic and non-eosinophilic phenotypes

**DOI:** 10.1590/0100-3984.2021.0088

**Published:** 2022

**Authors:** Rebecca Saray Marchesini Stival, Lêda Maria Rabelo, Giovanna Lemes Leão, Diogo Drevenowski, Joel Serafini, Vítor Lopes Galvão Vieira, Dante Luiz Escuissato

**Affiliations:** 1 Complexo Hospital de Clínicas, Universidade Federal do Paraná (UFPR), Curitiba, PR, Brazil

**Keywords:** Pulmonary disease, chronic obstructive, Eosinophils, Tomography, X-ray computed, Doença pulmonar obstrutiva crônica, Eosinófilos, Tomografia computadorizada

## Abstract

**Objective:**

To perform a quantitative assessment of bronchial wall thickening and the emphysema score in patients with stable chronic obstructive pulmonary disease (COPD), comparing the eosinophilic and non-eosinophilic COPD phenotypes.

**Materials and Methods:**

This was a retrospective observational study of patients with COPD followed between August 2018 and July 2019. The patients were divided into two groups by the eosinophil count in peripheral blood: eosinophilic (≥ 300 cells/µL); and non-eosinophilic (< 300 cells/µL). Quantitative, automated assessments of emphysema and bronchial wall thickness were performed by evaluating computed tomography scans of the chest.

**Results:**

We evaluated the records of 110 patients diagnosed with COPD: 28 (25.5%) in the eosinophilic group; and 82 (74.5%) in the non-eosinophilic group. The demographic, clinical, functional, and therapeutic variables were comparable between the two groups. There were no significant differences between the two groups in terms of the emphysema score or bronchial wall thickness (*p* > 0.05 for both).

**Conclusion:**

Patients with eosinophilic COPD do not appear to have lower emphysema scores or greater bronchial wall thickening than do those with non-eosinophilic phenotypes of the disease.

## Abstract (pt)

**Objetivo:**

Avaliar quantitativamente o escore de enfisema e o espessamento da parede brônquica de pacientes com doença pulmonar obstrutiva crônica (DPOC) estável e comparar os fenótipos eosinofílico e não eosinofílico.

**Materiais e Métodos:**

Estudo observacional, transversal, retrospectivo, que avaliou pacientes com DPOC no período de agosto de 2018 a julho de 2019. Os pacientes foram separados dois grupos, de acordo com o número de eosinófilos periféricos: os eosinofílicos (≥ 300 células/µL) e os não eosinofílicos (< 300 células/µL). Foram realizadas avaliações quantitativas e automatizadas de enfisema e de espessamento brônquico para os dois grupos por meio de tomografia computadorizada de tórax.

**Resultados:**

Foram coletados dados de 110 pacientes com o diagnóstico de DPOC, dos quais 28 (25,5%) apresentaram perfil eosinofílico. As variáveis demográficas, clínicas, funcionais e terapêuticas do grupo dos pacientes com perfil eosinofílico foram semelhantes às do grupo não eosinofílico. Não se observaram diferenças significativas em relação ao escore de enfisema e à medida de espessura de parede brônquica entre os dois grupos (*p* > 0,05).

**Conclusão:**

Neste estudo, os pacientes com fenótipo eosinofílico não apresentaram menor escore de enfisema e nem maior espessamento parietal brônquico.

## INTRODUCTION

Chronic obstructive pulmonary disease (COPD) is characterized by persistent lung airflow limitation that is not completely reversible, being diagnosed on the basis of spirometric criteria and a history of exposure to harmful particles or gases^(1)^. The diagnostic criteria have recently been a topic of discussion, and computed tomography (CT) of the chest is considered important to complete the tetrad for the diagnosis of COPD^(2)^: clinical manifestations; exposure; spirometric parameters; and CT findings. The current treatment for COPD is based on the use of bronchodilators and has the objective of decreasing lung hyperinflation, relieving symptoms, and reducing exacerbations^(1,3)^. There is a need to identify a biological marker that is able to predict the response to inhaled corticosteroids (ICS), which could inform the decision-making process regarding the timing of the initiation of ICS treatment in COPD patients with exacerbations, as well as regarding the discontinuation of ICS treatment when its side effects outweigh its benefits.

The eosinophil count in peripheral blood is a good indicator of the need for corticosteroid therapy^(4-6)^, having recently become an important biological marker for therapeutic decision-making. The 2019 Global Initiative for Chronic Obstructive Lung Disease (GOLD) report considers an eosinophil count ≥ 300 cells/µL in the first assessment of patients with COPD to be a criterion for the initiation of ICS^(1)^. It remains unclear whether eosinophilic inflammation of the airways, present in approximately 10-40% of patients with COPD^(7)^, plays a role in bronchial remodeling, which manifests as bronchial wall thickening on chest CT. It is also unknown if there is a direct relationship between eosinophilia and the amount of emphysema observed on imaging. Although COPD is a heterogeneous disease with an idiosyncratic progression, there have been few studies evaluating the CT profile of patients with eosinophilic COPD. Therefore, the objective of this study was to perform a quantitative assessment of the CT findings of emphysema and bronchial wall thickening in patients with stable COPD, as well as to evaluate their association with peripheral eosinophilia, defined as an eosinophil count ≥ 300 cells/µL.

## MATERIALS AND METHODS

This was a retrospective, cross-sectional observational study. Using the 2019 the GOLD report criteria, we assessed adult patients with COPD followed at the Pulmonology Outpatient Clinic of the Complexo Hospital de Clínicas da Universidade Federal do Paraná (CHC-UFPR, Federal University of Paraná Hospital de Clínicas Complex), in the city of Curitiba, Brazil. The CHC-UFPR Human Research Ethics Committee approved the study (Reference no. 01372618.0.0000.0096).

All of the patients were clinically stable. Exacerbation profiles were determined with the ABCD assessment tool, which was developed by the GOLD as a means of assessing the stage and severity of COPD^(1)^. We evaluated the eosinophil counts in peripheral blood measured in the most recent complete blood count. We also analyzed the most recent chest CT scan performed at the CHC-UFPR. Patients with asthma, asthma-COPD overlap syndrome, or alpha-1 antitrypsin deficiency were excluded. The study was conducted from 1 August, 2018 to 31 July, 2019, and the patients were divided into two groups according to the absolute number of eosinophils in their complete blood count: eosinophilic (≥ 300 cells/µL); and non-eosinophilic (< 300 cells/µL). The cutoff value to characterize the eosinophilic phenotype was 300 cells/µL.

The CT data were collected from high-resolution chest CT scans on file in the Picture Archiving and Communication System of the CHC-UFPR Radiology Department and analyzed at the Diagnóstico Avançado por Imagem clinic, also in the city of Curitiba. The chest CT scans were obtained in a 64-slice scanner (Aquillion; Toshiba Medical Systems, Tokyo, Japan), during full inspiration, at a tube voltage of 120 kVp and a tube current of 120 mAs. The images were reconstructed by using a soft-tissue convolution kernel (FC13) and 1-mm isotropic voxels. In all of the scans, we analyzed the presence of motion artifacts and whether or not all lung segments were included.

**Figure 1:**
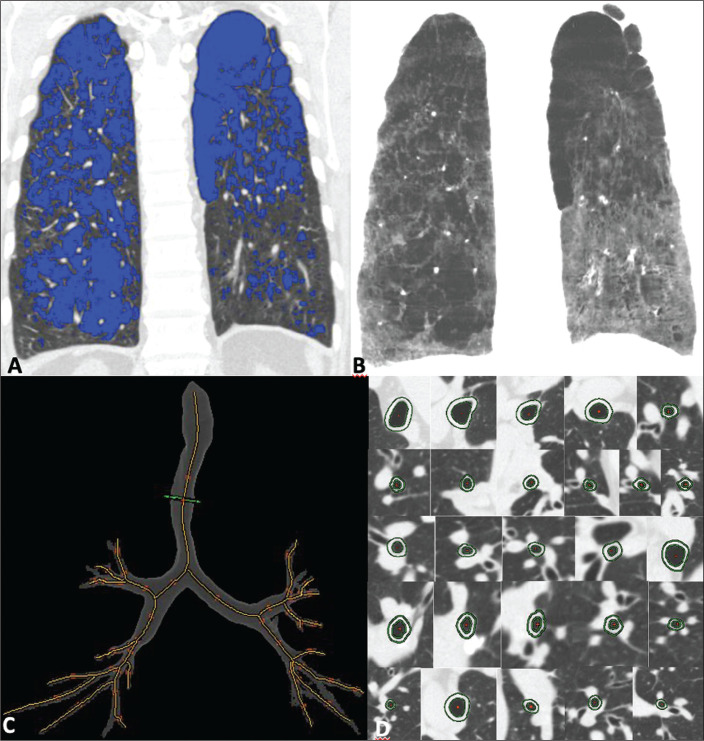
A,B: Quantification of emphysema on chest CT scans. A: Coronal image showing blue areas corresponding to regions with less than -950 HU (emphysema). B: Coronal image with minimum intensity projection reconstruction showing extensive areas of emphysema with low attenuation. C,D: Quantification of tracheal and bronchial wall thickness. C: Three-dimensional reformatting with lines on the central regions of the bronchial segments analyzed. D: Example of transversal plane measurement for each of the 27 segments (trachea, plus first, second, and third generation bronchi).

Qualitative image processing was performed on a standard workstation (AW server; GE Healthcare, Milwaukee, WI, USA) with thoracic volume computer assisted reading software (GE Healthcare), which allowed automated lung segmentation and automated measurement of bronchial wall thickness. After the lung had been segmented, we quantified the degree of emphysema by determining the attenuation values-in Hounsfield units (HU)-, calculating the proportion of areas with attenuation below a predetermined threshold (Figure 1). As in several other studies, the threshold selected for the present study was -950 HU, any area with an attenuation value below that threshold being categorized as emphysematous^(8,9)^. We also calculated the lung volumes and proportional areas of emphysema.

The airways were analyzed by semi-automated segmentation^(10)^. After selecting the bronchus for analysis, the software reconstructed and rectified the airway, enabling the measurement of the wall thickness (Figure 1). The selection included measurements of the following: trachea; main bronchi; upper lobe bronchi; left lower lobe bronchus; right, lower, and middle bronchi; and segmental bronchi (three right upper lobe segments, two middle lobe segments, four left upper lobe segments, five right lower lobe segments, and four left lower lobe segments). In each of those locations, the measurements were made in the middle third of the segment of interest or the spot at which the automated segmentation had shown the wall thickness to be most representative of the mean wall thickness, as determined by a radiologist with five years of experience in thoracic radiology. In all 110 patients, the bronchi were scanned down to the third generation. Scans with segmentation or quantification errors, as determined by the radiologist, were excluded from the analysis.

### Statistical analysis

Continuous variables are presented as means and standard deviations (SDs), with minimum and maximum values, whereas categorical variables are presented as absolute and relative frequencies. For between-group comparisons of categorical variables, Fisher’s exact test or the chi-square test was used. For continuous variables, the groups were compared by using the Student’s t-test for independent samples or the nonparametric Mann-Whitney U test. The Kolmogorov-Smirnov test was used in order to assess the normality of the continuous variables. Values of *p* < 0.05 were considered indicative of statistical significance. Statistical analyses were performed with the IBM SPSS Statistics software package, version 20.0 (IBM Corp., Armonk, NY, USA), as described by Pagano et al.^(11)^.

## RESULTS

Of the 386 patients with COPD followed during the study period, 110 (28.5%) met the inclusion criteria. Among those 110 patients, the eosinophil count in peripheral blood was ≥ 300 cells/µL in 28 (25.5%) and < 300 cells/µL in 82 (74.5%), those patients therefore composing the eosinophilic and non-eosinophilic groups, respectively. The demographic, functional, and clinical characteristics of the patients were similar between the two groups (Tables 1 and 2), demonstrating that the sample was homogeneous. As assessed with the ABCD tool^(1)^, 36 (43.9%) of the 82 patients in the non-eosinophilic group had an exacerbation profile, compared with 10 (35.7%) of the 28 patients in the eosinophilic group. Of the patients in the non-eosinophilic group, 52 (63.4%) used ICS, as did 19 (67.9%) of those in the eosinophilic group.

**Table 1:** Baseline demographic and clinical characteristics of patients with eosinophilic and non-eosinophilic COPD.^*^

Variable	All patients (N = 110)	Group		*P* ^†^
		Eosinophilic (n = 28)	Non-eosinophilic (n = 82)	
Age (years)	67.4 ± 9 (38-87)	69.3 ± 11.6 (42-87)	66.8 ± 8 (38-87)	0.294
Sex, n (%)				
Female	62 (56.4)	15 (53.6%)	47 (57.3%)	
Male	48 (43.6)	13 (46.4%)	35 (42.7%)	0.826
White, n (%)	97 (88.2)	23 (82.1)	74 (90.2)	
Body mass index (kg/m^2^)	25.8 ± 5.9 (12.3-48)	25.1 ± 6.5 (12.3-48)	26.1 ± 5.6 (15.2-8.7)	0.422
GOLD group^‡^, n (%)				
A	25 (22.7)	7 (25)	18 (22)	
B	39 (35.5)	11 (39.3)	28 (34.2)	
C	6 (5.5)	0 (0)	6 (7.3)	
D	40 (36.4)	10 (35.7)	30 (36.6)	0.514
GOLD grade^§^, n (%)				
1	8 (7.3)	2 (7.1)	6 (7.3)	
2	42 (38.2)	11 (39.3)	31 (37.8)	
3	36 (32.7)	9 (32.1)	27 (32.9)	
4	24 (21.8)	6 (21.4)	18 (22)	0.999
Peripheral oxygen saturation (%)	92.1 ± 4.5 (77-98)	92 ± 4.5 (80-98)	92.1 ± 4.6 (77-98)	0.910
Current smoking, n (%)	23 (20.9)	6 (21.4)	17 (20.7)	1
Smoking history (pack-years)	53 ± 31.2 (0-155)	60.2 ± 39.8 (0-155)	50.5 ± 27.6 (1-150)	0.291

* Values expressed as mean ± SD (range), except where otherwise indicated.

† Student’s t-test for independent samples, Fisher’s exact test, or chi-square test.

‡ Groups A and B include patients who have had no exacerbations or one previous exacerbation that did not require hospital admission, respectively; groups C and D include patients who have had two or more exacerbations or at least one exacerbation requiring hospital admission, respectively, in the previous year.

§ The number provides information regarding the severity of airflow limitation: grade 1 = forced expiratory volume in 1 second (FEV_1_) ≥ 80% of predicted; grade 2 = FEV_1_ 50-79% of predicted; grade 3 = FEV_1_ 30-49% of predicted; and grade 4 = FEV_1_ < 30% of predicted.

**Table 2:** Clinical characteristics of patients with eosinophilic and non-eosinophilic COPD.^*^

Variable	All patients (N = 110)	Group		*P* ^†^
		Eosinophilic (n = 28)	Non-eosinophilic (n = 82)	
Moderate exacerbations without hospitalization^‡^ (n)	0.5 ± 0.9 (0-5)	0.4 ± 0.9 (0-4)	0.5 ± 0.9 (0-5)	0.621
0-1, n (%)	99 (90.0)	27 (96.5)	72 (87.8)	
≥ 2, n (%)	11 (10.0)	1 (3.6)	10 (12.1)	
Severe exacerbations with hospitalization^‡^ (n)	0.3 ± 0.6 (0-3)	0.4 ± 0.7 (0-3)	0.4 ± 0.7 (0-3)	0.835
≥ 1, n (%)	29 (26.3)	8 (28.6)	21 (25.6)	
Low-dose ICS^§^ (budesonide < 800 µg)				1
No, n (%)	88 (80.0)	23 (82.1)	65 (79.3)	
Yes, n (%)	22 (20.0)	5 (17.9)	17 (20.7)	
High-dose ICS^§^ (budesonide ≥ 800 µg)				0.517
No, n (%)	61 (55.5)	14 (50.0)	47 (57.3)	
Yes, n (%)	49 (44.5)	14 (50.0)	35 (42.7)	

* Values expressed as mean ± SD (range), except where otherwise indicated.

† Student’s t-test for independent samples, nonparametric Mann-Whitney U test, chi-square test, or Fisher’s exact test.

‡ In the last year before data collection.

§ At the time of data collection.

For the analysis of bronchial wall thickness, the images obtained for nine patients could not be evaluated, because of errors in airway segmentation, three patients having been excluded from the analysis because of difficulties in measuring the thickness of some segmental bronchi, due to beam hardening artifacts or anatomical variations. Therefore, data regarding the thickness of the trachea, main bronchi, and segmental bronchi were available for only 101 patients. Tables 3 and 4 show those data for the right and left lungs, respectively. There was no statistically significant difference between the two groups in terms of the mean thickness of any given bronchial wall segment or the overall mean bronchial wall thickness (*p* > 0.05 for all).

**Table 3:** Comparison of tracheal and bronchial wall thickness (in mm) in the right lung between patients with eosinophilic and non-eosinophilic COPD.^*^

Segment	Patients evaluated (n)	All patients (N = 101)^†^	Group		*P* ^‡^
			Eosinophilic (n = 25)	Non-eosinophilic (n = 76)	
Trachea	101	3.1 ± 0.5 (2.3-4.9)	3.0 ± 0.4 (2.3-3.8)	3.1 ± 0.6 (2.3-4.9)	0.180
MRB	101	3.0 ± 0.5 (2.2-4.3)	2.9 ± 0.4 (2.2-3.8)	3.0 ± 0.5 (2.2-4.3)	0.451
RUL	101	2.6 ± 0.4 (1.7-3.5)	2.6 ± 0.4 (2.0-3.5)	2.7 ± 0.4 (1.7-3.4)	0.346
Ant-RUL	101	2.2 ± 0.3 (1.2-3.2)	2.3 ± 0.4 (1.6-3.1)	2.2 ± 0.3 (1.2-3.2)	0.305
Api-RUL	101	2.1 ± 0.4 (1.4-3.2)	2.2 ± 0.4 (1.5-3.2)	2.1 ± 0.4 (1.4-3.2)	0.742
Pos-RUL	101	2.1 ± 0.3 (0.9-2.8)	2.1 ± 0.3 (1.6-2.8)	2.1 ± 0.3 (0.9-2.8)	0.917
ML	100	2.4 ± 0.4 (1.7-4.3)	2.3 ± 0.3 (1.7-2.8)	2.5 ± 0.4 (1.7-4.3)	0.074
M-ML	99	2.1 ± 0.4 (1.4-3.2)	2.2 ± 0.4 (1.4-3.1)	2.1 ± 0.4 (1.4-3.2)	0.244
L-ML	99	2.1 ± 0.4 (1.1-3.1)	2.1 ± 0.4 (1.3-3.1)	2.0 ± 0.4 (1.1-3.1)	0.324
RLL	100	2.6 ± 0.4 (1.9-4.0)	2.5 ± 0.4 (1.9-3.7)	2.6 ± 0.5 (1.9-4.0)	0.280
U-RLL	99	2.3 ± 0.4 (1.5-3.5)	2.3 ± 0.3 (1.9-3.0)	2.3 ± 0.4 (1.5-3.5)	0.838
AB-RLL	99	2.2 ± 0.3 (1.3-3.0)	2.2 ± 0.3 (1.5-3.0)	2.2 ± 0.3 (1.3-2.8)	0.930
PB-RLL	99	2.1 ± 0.3 (1.5-2.9)	2.1 ± 0.3 (1.7-2.7)	2.1 ± 0.3 (1.5-2.9)	0.963
MB-RLL	98	2.0 ± 0.3 (1.4-2.9)	2.0 ± 0.3 (1.5-2.7)	2.0 ± 0.3 (1.4-2.9)	0.632
LB-RLL	99	2.2 ± 0.3 (1.5-3.1)	2.2 ± 0.3 (1.5-3.0)	2.2 ± 0.3 (1.6-3.1)	0.489

* Values expressed as mean ± SD (range).

† In nine patients, bronchial thickness could not be evaluated, because of errors in airway segmentation.

‡ Student’s t-test for independent samples.

MRB, main right bronchus; RUL, right upper lobe bronchus; Ant-RUL, anterior bronchus of the RUL; Api-RUL, apical bronchus of RUL; Pos-RUL, posterior bronchus of the RUL; ML, middle lobe bronchus; M-ML, medial bronchus of the ML; L-ML, lateral bronchus of the ML; RLL, right lower lobe bronchus; U-RLL, upper bronchus of the RLL; AB-RLL, anterior basal bronchus of the RLL; PB-RLL, posterior basal bronchus of the RLL; MB-RLL, medial basal bronchus of the RLL; LB-RLL, lateral basal bronchus of the RLL.

**Table 4:** Comparison of bronchial wall thickness (in mm) in the left lung between patients with eosinophilic and non-eosinophilic COPD, together with the overall bronchial wall thickness for both lungs.^*^

Segment	Patients evaluated (n)	All patients (N = 101)^†^	Group		*P* ^‡^
			Eosinophilic (n = 25)	Non-eosinophilic (n = 76)	
MLB	101	3.0 ± 0.5 (2.1-4.4)	3.0 ± 0.5 (2.2-3.8)	3.0 ± 0.5 (2.1-4.4)	0.474
LUL	101	2.9 ± 0.5 (1.8-5.0)	2.7 ± 0.4 (1.8-3.7)	2.9 ± 0.6 (1.8-5.0)	0.084
Ant-LUL	100	2.2 ± 0.4 (0.9-3.2)	2.2 ± 0.4 (0.9-3.2)	2.3 ± 0.3 (1.6-3.1)	0.530
Api-Pos-LUL	100	2.2 ± 0.4 (1.4-3.2)	2.3 ± 0.4 (1.4-3.1)	2.2 ± 0.3 (1.6-3.2)	0.393
LgL	101	2.4 ± 0.4 (1.7-3.8)	2.4 ± 0.4 (1.9-3.5)	2.5 ± 0.4 (1.7-3.8)	0.844
U-LgL	99	2.0 ± 0.3 (1.3-2.8)	2.0 ± 0.3 (1.5-2.7)	2.0 ± 0.3 (1.3-2.8)	0.991
L-LgL	100	2.0 ± 0.3 (1.3-2.8)	2.0 ± 0.2 (1.3-2.4)	2.0 ± 0.3 (1.3-2.8)	0.386
LLL	100	3.0 ± 0.5 (2.0-4.4)	2.9 ± 0.5 (2.0-3.9)	3.0 ± 0.5 (2.0-4.4)	0.265
U-LLL	100	2.3 ± 0.3 (1.4-3.1)	2.3 ± 0.4 (1.4-3.1)	2.3 ± 0.3 (1.7-3.1)	0.999
AMB-LLL	100	2.2 ± 0.3 (1.5-3.4)	2.3 ± 0.3 (1.5-3.4)	2.2 ± 0.3 (1.6-3.0)	0.413
PB-LLL	100	2.2 ± 0.3 (1.6-3.2)	2.2 ± 0.3 (1.6-3.0)	2.2 ± 0.3 (1.7-3.2)	0.647
LB-LLL	99	2.3 ± 0.3 (1.6-3.7)	2.3 ± 0.4 (1.7-3.0)	2.3 ± 0.4 (1.6-3.7)	0.680
Overall (both lungs)	101	2.4 ± 0.2 (2.0-2.9)	2.4 ± 0.2 (2.0-2.8)	2.4 ± 0.2 (2.0-2.9)	0.802

* Values expressed as mean ± SD (range).

† In nine patients, bronchial thickness could not be evaluated, because of errors in airway segmentation.

‡ Student’s t-test for independent samples or nonparametric Mann-Whitney U test.

MLB, main left bronchus; LUL, left upper lobe bronchus; Ant-LUL, anterior bronchus of the LUL; Api-Pos-LUL, apico-posterior bronchus of the LUL; LgL, lingular lobe bronchus; U-LgL, upper lingular bronchus; L-LgL, lower lingular bronchus; LLL, left lower lobe bronchus; U-LLL, upper basal bronchus of the LLL; AMB-LLL, anteromedial basal bronchus of the LLL; PB-LLL, posterior basal bronchus of the LLL; LB-LLL, lateral basal bronchus of the LLL.

Table 5 shows the lung volumes and proportional areas of emphysema, by group. In two patients, the quantitative analysis of emphysema was not possible, because of architectural distortion of the lung parenchyma. Therefore, data regarding the volume and proportional area of emphysema were available for only 108 patients. Nevertheless, there was no statistically significant difference between the two groups in terms of the mean lung volumes (*p* > 0.05 for all).

**Table 5:** Quantitative comparison of emphysema between patients with eosinophilic and non-eosinophilic COPD.^*^

Variable	All patients (N = 108)^†^	Group		*P* ^‡^
		Eosinophilic (n = 27)	Non-eosinophilic (n = 81)	
RL Vol. (L)	2.9 ± 0.7 (0.8-5.1)	2.9 ± 0.8 (0.8-4.2)	2.9 ± 0.7 (1.7-5.1)	0.965
LL Vol. (L)	2.5 ± 0.7 (0.8-4.6)	2.5 ± 0.8 (0.8-3.9)	2.5 ± 0.6 (1.3-4.6)	0.920
Total Vol. (L)	5.4 ± 1.3 (2.2-9.0)	5.4 ± 1.6 (2.2-8.1)	5.4 ± 1.3 (2.5-9.0)	0.874
RL Emph. %	26 ± 19 (1-94)	23 ± 18 (1-58)	26 ± 20 (1-94)	0.471
LL Emph. %	22 ± 18 (0-91)	21 ± 17 (0-58)	23 ± 19 (1-91)	0.587
Total Emph. %	24 ± 18 (0-91)	22 ± 17 (1-58)	24 ± 18 (0-91)	0.528
RL Emph. Vol. (L)	0.71 ± 0.64 (0.01-2.41)	0.71 ± 0.64 (0.01-2.41)	0.82 ± 0.72 (0.01-3.51)	0.456
LL Emph. Vol. (L)	0.58 ± 0.60 (0-2.29)	0.58 ± 0.60 (0-2.29)	0.63 ± 0.63 (0.01-3.26)	0.708
Total Emph. Vol. (L)	1.29 ± 1.22 (0.02-4.71)	1.29 ± 1.22 (0.02-4.71)	1.40 ± 1.23 (0.01-6.41)	0.702

* Values expressed as mean ± SD (range).

† In two patients, emphysema could not be quantified, because of architectural distortion of the lung parenchyma.

‡ Student’s t-test for independent samples or nonparametric Mann-Whitney U test.

RL Vol., right lung volume; LL Vol., left lung volume; Total Vol., total lung volume; RL Emph. %, right lung emphysema percentage; LL Emph. %, left lung emphysema percentage; Total Emph. %, total emphysema percentage; RL Emph. Vol., right lung emphysema volume; LL Emph. Vol., left lung emphysema volume; Total Emph. Vol., total emphysema volume.

## DISCUSSION

The eosinophil count has been increasingly considered to be a biological marker of COPD, especially after its inclusion in the algorithm of the 2019 GOLD treatment guidelines. The eosinophil count in peripheral blood became a criterion for the initiation and discontinuation of ICS therapy in patients with stable COPD, as well as for therapeutic management of COPD with exacerbations, as recommended in the 2019 GOLD report^(1)^. That has been supported by previous studies such as the Withdrawal of Inhaled Steroids During Optimized Bronchodilator Management study^(12)^, in which it was observed that the discontinuation of ICS therapy in patients with an eosinophil count ≥ 300 cells/µL increased the risk of exacerbation^(13)^. However, other significant studies, such as that conducted by Ferguson et al.^(14)^, used an eosinophil count cutoff of 150 cells/µL, which was found to have a positive impact on ICS therapy in patients with eosinophilic COPD^(15)^.

In patients with COPD, the pathophysiology of airway inflammation is predominantly neutrophilic. Nevertheless, up to one third of the patients present with eosinophilic airway inflammation. That profile is seen more commonly in patients in whom the predominant manifestation is chronic bronchitis than in those in whom it is emphysema^(16)^. Eosinophils stimulate the release of cytokines, chemokines, and leukotrienes that could be responsible for bronchial remodeling, given that, in patients with asthma, there is a relationship between eosinophilia and bronchial thickening^(17)^. Our finding that there was no association between the eosinophil count in peripheral blood and bronchial wall thickening could be due to the fact that the frequency of ICS use was high in our sample: 67.9% in the eosinophilic group and 63.4% in the non-eosinophilic group. The fact that 43.9% of the patients in our non-eosinophilic group had an exacerbation profile, as did 35.7% of those in our eosinophilic group, together with the fact that the frequency of ICS use was high in both groups, could explain our difficulty in establishing a significant difference between the two groups in terms of the chest CT findings.

Our study has some limitations. First, because of the retrospective study design, the data were collected exclusively from medical records. In addition, the data were obtained by convenience sampling. Although we were careful in selecting the blood counts obtained closest to the date of the imaging, it is known that the eosinophil count can fluctuate from one measurement to another. However, our high cutoff point for characterizing the eosinophilic phenotype might have mitigated such variations. As previously mentioned, the precise cutoff point to identify patients with eosinophilic COPD has yet to be established. Our choice of an eosinophil count in peripheral blood of 300 cells/µL as the cutoff point might also be construed as a limiting factor. In a previous study evaluating the relationship between peripheral eosinophils and emphysema^(18)^, the authors showed that patients with significant emphysema observed on chest CT scans had low eosinophil counts in peripheral blood (mean, 34.6 cells/µL) in comparison with the patients with no significant emphysema (mean, 169 cells/µL), although no patients with an eosinophil count over 300 cells/µL were included in that study. In the present study, we did not observe a relationship between peripheral eosinophilia and the quantitative emphysema score, as evaluated by chest CT.

Further studies, especially prospective studies, are warranted in order to evaluate the clinical, functional, and CT characteristics of patients with eosinophilic COPD. That could help establish the best cutoff point to define this population and thus enable the use of treatments that are more aggressive.
